# Discussion on the Pension Fund Paper

**Published:** 1887-10-22

**Authors:** 


### Discussion on the Pension Fund Paper.

The Chairman: This is one of the most interesting as well as one of the most important papers read before The Hospitals Association. The time, the labour, the thought, the ability which Mr. Burdett has devoted to the subject will, I am certain, obtain for him your most cordial thanks. The paper has necessarily occupied a long time, and I will not shorten the period remaining by saying much myself, but I will recall to your minds the principles and the broad statements which have been made in the paper, because their remembrance may be a guide to you. Before I mention those broad points in the paper, allow me to state one thing to supplement what Mr. Burdett has said why you should have a Fund of your own, and not go to the Post-office or any other existing institution. The first reason is this: that many people before they are long upon the Fund will die, and there will be a surplusage which will go to the benefit of others. In the next place, when this Fund is started, it will receive benevolent contributions, not only from hospitals and committees of management, but from individuals who may have benefited from the services of nurses; and the third and last reason is, that nine-tenths of any expenses incurred in the administration of the Fund would be defrayed by voluntary services. The points which Mr. Burdett has shown are these: First, that there is an enormous body of nurses, to the number of about 15,000, from which members may be drawn. Secondly, that the nature of their work is such that they are peculiarly liable to accidents and diseases, to premature inability to work, and to premature old age, to a degree not experienced by other workers. In the third place he has shown that no adequate provision has as yet been made to give pensions to these workers, or to supply them with help in time of sickness. He has shown that, from these considerations and others, it is the duty, not only of nurses themselves, but of society and of hospitals also, that a National Pension Fund should be established. In the next place he has shown that the first condition in the establishment of such a Fund is that it should be financially sound and self-supporting. Independently of whatever may be given to it from outside, it should be financially capable of standing by itself. This is a golden condition. Then he has suggested that, as Her Majesty the Queen, with that gracious consideration for her people which has distinguished her reign, is about to apply for the benefit of nurses and nursing institutions £75,000, which her country has offered her as a memorial of her Jubilee, application should be made for a portion of that fund sufficient to proceed at once to action. For my own part, I doubt if this question can be profitably discussed to-night, but I presume that this meeting will not end here, but that some sort of a committee will be formed, and meet over a table and discuss these things in a familiar and informal way. I shall now ask some one who is interested in this question to open the discussion.

Mr. P. Michelli, Secretary, Seamen's Hospital, Greenwich: This is certainly a most interesting paper. It is worthy of Mr. Burdett, who, we know, has such matters at his fingers' ends more than any one else in London. I would like to point out one or two things. Mr. Burdett brought out very plainly that a payment of 12s. 6d. per annum towards a sick-fund, which has no profits, will realise to that person a very considerable amount when sick and disabled. I agree with what Miss Vincent says in the letter which Mr. Burdett has just read, that more may be done in this way. With regard to pensions, a sum of £12 or £15 a year seems to me very little. I would prefer to see her paying her £3 5s. in the spirit of the India Fund, which pays such large pensions. That is the system on which, I think, we ought to go. Our Chairman has said that he hopes this matter will be adjourned to a committee. I rather hope not. To committee, we all know, it must go eventually, but now I think we want to hear more opinions. There are many who have not been able to come here to-night who are in full sympathy with this Fund. If a meeting is again convened we shall doubtless have a large gathering.

Mr. Geo. King, Actuary to the Atlas Office, Cheapside: I am very much obliged to Mr. Burdett for having asked me to come to this meeting. It has interested me both as an actuary and as a member of a community to whom hospitals and nurses are so valuable. Mr. Burdett's paper has been exhaustive, and has dealt with the question in a most lucid manner. With your permission, I would like just to make one or two remarks. In the first place, I would remind you that the pensions which Mr. Burdett has mentioned are minimum, not maximum pensions. Mr. Burdett said it was necessary to place the Fund on a satisfactory basis, and you can only do that by fixing minimum rates. The Chairman has mentioned the surplusage accruing to the Fund from the contributions of those who die or retire, but until it is known how many will retire it would not be safe to deal with it. The Fund will have to be valued for three or five years, and at the end of this period any surplus fund will be available for distribution. Then it will go to those who remain with the Fund, increasing the benefits or reducing the contributions of those who are its steadfast friends. Further, I may say that the calculations have been made entirely on the supposition that the Fund is self-supporting, but unquestionably large sums will be available from donations. These will all be equitably applied to increase the benefits or diminish the contributions of members, and will doubtless form a very substantial source of increased benefits to those who stand by the Fund. If Her Gracious Majesty should give the sum which it is proposed to ask for to establish the Fund, the interest upon it would be valuable, amounting to, say, from £600 to £1,000 per annum, and that would be at once available to increase the benefits or reduce the contributions of subscribers. In connection with the matter of donations, let it be remembered that they are not charity, but merely pay in another form. The Fund does not look for charity at all. In concluding, I would urge you, Don't ask for what is impossible. There is a limit, and by calculation we find what can be done. Let us, therefore, determine to carry out what is possible. If all those who are interested in this matter will only make up their minds to carry out what can be done, a most prosperous and successful Fund will be established.

Miss Ingall, matron of the London Fever Hospital, said she had been thinking of trying to arrange a fund at her institution in which each member should pay a percentage of her salary for a pension. She thought that those who had large salaries might help those who had small ones. With reference to individuals who, in return for a nurse's attention, might give her a donation, that sum, she thought, ought to go to a general fund, and not to the nurse personally.

Lord Sandhurst, Vice-President of the Middlesex Hospital: I feel, Sir Andrew, that in addressing this audience my remarks are entirely confined to ladies, and to ladies who know very much more of the subject we are discussing than I can pretend to know. I came here more as a listener than to take an active part in these proceedings. I have for some time, however, taken a great interest in work of this description. I take also an interest in one of the largest London hospitals, and am a member of The Hospitals Association. I think it is somewhat to be regretted that we have not been able to get more suggestions of a practical kind than the ones to which we have just listened. In such an audience as this there are those who must know of the sufferings of their old colleagues, and we might have looked for some advice from them; but I am hopeful that we shall receive advice, for I confess that the figures and the question generally, although admirably explained by Mr. Burdett, are of a complex nature. Indeed, it took all my best attention to understand some of them. Allusion has been made to the fund at the disposal of the Queen—the Women's Jubilee Offering. It seems to me that there is no more fitting way in which the Queen could have acknowledged that gift than by returning it, as she is doing, and giving it to a charitable object such as has been described. It has been said, I think by Mr. Burdett, that a committee has been appointed to consider the best way in which that sum may be dealt with in support of nurses for pensions and for other means. That I think is a far more graceful way of acknowledging the gift of the women of England than if Her Majesty had built some great hall which, although pleasurable for those who could reach such a place easily, could not confer such general benefit as the application of the Fund for the good of nurses. There is another way to look at the benefits which nursing has done for us. What was in former days to be done for a pound is now to be done for a shilling. There are many cases in which the patient has been brought to health by careful nursing. That is one of the principal needs in typhoid fever, a disease of which I have some experience. I am perfectly certain that health has been restored in some cases by the unceasing labour on the part of the nurses employed. I say this in no way because I am addressing an assemblage of nurses, or of ladies principally interested in nursing, but what I say I know to be actual fact, and I am excessively glad to have been here to-night to listen to the admirable lecture given us by Mr. Burdett, a gentleman who has given many years and great thought to a most difficult subject, and I have been asked to propose a resolution which I think meets the case. It is—"That the principles of the scheme of Mr. Burdett be generally approved by this meeting, and that a copy of the paper now read be forwarded to Sir Henry Ponsonby for the consideration of Her Majesty's Committee that has been appointed for the distribution of the Fund at Her Majesty's disposal."

Mr. THOMAS RYAN, Secretary, St. Mary's Hospital, in seconding the resolution, said: When this question of a Pension Fund first came under my notice, I was inclined to ask myself wherein lay the need for the Fund at all? A little consideration, however, brought me face to face with two reasons why it is necessary to establish a National Pension Fund. The first was this: that the provision that is made by the hospitals for pensioning some of their officials leaves out of consideration one very marked feature. I refer to the migration of nurses from one hospital to another. The point that hospitals do not provide for those who move from place to place is, I take it, an important one. Migration of nurses is a feature of hospital life which is growing very rapidly. I have recently had occasion to look into the question of pensions. Dr. Steele said this moving of nurses from place to place was quite a recent feature. Time was when they used to pass their whole career in one institution. Now, if a nurse passed her whole life in one institution she would be granted a pension, but five or six years' service will not give her a claim to a pension, and that is the very feature which this Pension Fund will meet. The Trained Nurses' Annuity Fund does not meet the necessities of the case. I will now turn to the financial aspect of the question. Mr. Burdett has explained that nurses leaving nursing should be able to draw out either all or some portion of the money they have paid in. In that case the only funds left for pensioning those who remain are their own contributions. I am convinced of the absolute necessity which exists for an Endowment Fund, and I think the public might be called upon to contribute to this. The capital fund of The Home Hospitals Association, which was, I believe, originated by Mr. Burdett himself, was provided at the cost of the general public. That the Queen may be very graciously pleased to grant a very considerable portion of the Women's Jubilee Offering to this Fund, I do sincerely hope, and I think that the hospitals also should contribute as a bounden duty.

The resolution having been put from the chair, was unanimously carried.

Mr. BURDETT: I should like, instead of leaving it to another evening, that this meeting, which is so large and so representative, should not break up without coming to some practical conclusion itself, and that it should, as a meeting, elect some representative body to represent it and put the necessary machinery in motion in order to carry the scheme to a successful issue. I beg therefore to move:

"That this meeting, representing 138 institutions from which 1,172 officials and nurses have registered their names in support of the scheme for founding a National Pension Fund for Hospital Officials and Trained Nurses, beg to refer the scheme to the Council of The Hospitals Association, and desire them to take such steps as may be necessary to establish this Fund with as little delay as possible."

I was very sorry to hear the remarks of Mr. Ryan with reference to an appeal to the public for help. I have known Mr. Ryan a good many years. He is a relatively young man, and therefore relatively enthusiastic. It is not a question of charity, but a question of providence; and if you have not got the providence and the self-help, I hope there will be no fund at all. We don't want, as we did in the case of the Home Hospitals, to get capital to build up something. If you, a great army of 20,000 working-beings, have not got self-reliance, energy, and independence enough to build up this Fund on a financially sound basis, believing that Providence will help you, all I can say is that the time is not ripe for it. It is for you to begin on what I tell you is a sound financial basis, entirely dependent upon your own contributions and your own efforts. I have told you that any nurse who contributes 14s. 6d. a year to this Fund will be entitled to 10s. per week sick-relief during her illness until she attains 60 years of age. I certainly do think that if every hospital nurse in this country knew this she would be grateful for it; if she is not, then I don't know the feeling of hospital nurses. So far as the second portion of the scheme goes, you pay your £2 10s. a year, and you will have a minimum pension at 60 and during life of from £12 to £15 per annum—that, I say, is done for you by the power of compound interest.

Mr. C. W. PEARCE, Associate of the Institute of Actuaries, Glasgow, seconded the resolution, which was also unanimously carried.

Dr. G. W. POTTER, in proposing that the best thanks of the meeting be given to Sir Andrew Clark, observed that it should be borne in mind that the minimum pension of £12 or £15 per annum left out of the question the interest which would be derived from the Queen's Bounty, or any contributions that friends might give. Those who paid their £2 10s., if they worked on till fifty-five or sixty, might rely upon a pension of 10s. a week. If a payment of £2 10s. a year brought them an independency at the age of sixty, he thought they ought to consider themselves very well off.

Col. MONTEFIORE seconded the vote of thanks to the Chairman, and the meeting terminated.

